# 2D association and integrative omics analysis in rice provides systems biology view in trait analysis

**DOI:** 10.1038/s42003-018-0159-7

**Published:** 2018-09-27

**Authors:** Wenchao Zhang, Xinbin Dai, Shizhong Xu, Patrick X. Zhao

**Affiliations:** 1 Computational Biology and Bioinformatics Lab, Noble Research Institute, Ardmore, OK 73401 USA; 2 Department of Botany and Plant Sciences, University of California, Riverside, CA 92521 USA

## Abstract

The interactions among genes and between genes and environment contribute significantly to the phenotypic variation of complex traits and may be possible explanations for missing heritability. However, to our knowledge no existing tool can address the two kinds of interactions. Here we propose a novel linear mixed model that considers not only the additive effects of biological markers but also the interaction effects of marker pairs. Interaction effect is demonstrated as a 2D association. Based on this linear mixed model, we developed a pipeline, namely PATOWAS. PATOWAS can be used to study transcriptome-wide and metabolome-wide associations in addition to genome-wide associations. Our case analysis with real rice recombinant inbred lines (RILs) at three omics levels demonstrates that 2D association mapping and integrative omics are able to provide a systems biology view into the analyzed traits, leading toward an answer about how genes, transcripts, proteins, and metabolites work together to produce an observable phenotype.

## Abstract (web summary)

Wenchao Zhang et al. developed a tool for analyzing traits using data generated from genome-wide, transcriptome-wide, and metabolome-wide association studies. They test their approach in rice, providing a systems biology view of identified traits.

## Introduction

Trait analysis, especially genome-wide trait analysis, is centered on how genetic variation gives rise to phenotypic variation^1^. This type of analysis relies on statistical methods and tools to perform association mapping between causal genetic variants and resulting phenotypes, which can determine the heritability of a trait at a subset of genetic variants (typically referred to as single-nucleotide polymorphisms, or SNPs) and delineate regions of the genome that control the trait, thereby providing markers that can be utilized to accelerate breeding by marker-assisted selection^2^. Because of the great success of genome-wide association studies (GWAS), hundreds of SNPs conferring genetic variation of complex traits have been identified and reported^3^. However, the genetic structures of most traits remain unexplained, as associated SNPs detected from GWAS explain only a small fraction of heritability (e.g., <40% in schizophrenia studies)^4^ and a much smaller percentage of the total phenotypic variance. This is mainly because a number of these studies employed only additive models that fail to account for epistasis, or the interaction between multiple loci and the environment^4–6^.

Xu et al.^7^ proposed a new linear mixed model (LMM) for mapping quantitative loci (QTL) by incorporating multiple polygenic covariance structures. Based on this model, a pipeline for estimating epistatic effects (PEPIS) was developed to comprehensively estimate additive effects, dominance effects, and interaction effects between multiple genetic loci. PEPIS allows analysis of genome-wide genetic architectures, including genotype interaction effects (GxG), and can thereby explain more than 80% of phenotypic variance^8^.

Compared with standard GWAS tools that consider only additive effects, the PEPIS pipeline is equipped with a more complex polygenic linear model that can explain more phenotypic variance. However, neither of these methods can explain nearly 100% of phenotypic variance, as neither considers the interaction between genotypes and environments (GxE). Today, the predominant thinking in biology is that the orchestrated expression of many genes in different environmental conditions affects the transcriptome, proteome, and metabolome to produce a final observable phenotype^9^. Recent work in *Saccharomyces cerevisiae* suggests that GxE can occur at the individual locus level and the group level for multiple loci, leading to environment-dependent epistatic interactions^10–12^. Although Muir et al.^13^ conceptualized the partitioning of GxE into two possible interaction types, our mathematical understanding of the genetic and molecular mechanisms by which GxE collectively gives rise to phenotypes is still incomplete^14^.

The central dogma of biology is that the genome, transcriptome, proteome, and metabolome are cascading and connected to the end phenome^15^. The development of life science technologies enables transcriptomic, proteomic, and metabolomic events to be analyzed in detail within the same biological system, allowing the systematic study of a complete biological system^16^. Out of all the omic data from the same biological system, genomic data generally remain constant across environments, although the same genotype subjected to different environments can produce a wide range of phenotypes by triggering the expressions of different genes, downstream enzymes, and metabolites^17^. Most current association methods and analysis tools perform association mapping based on fundamental relationships between DNA sequence variation and phenotypic variation without addressing environmental variation. GxE can be understood by observing and measuring the expression of genes or metabolites. Harper et al.^2^ developed an associative transcriptomic approach to study complex traits in the polyploidy crop species *Brassica napus* by correlating trait variation with the quantitative expression of genes and sequence variation of transcripts, with the consistent physical positions of the two kinds of associative markers allowing the identification of high-confidence transcription factor candidates^2,18^. However, their method is based on a pure additive model only, and they make no mention of interaction effects between biomarkers or their contribution to phenotypic variation.

To overcome the limitation of standard GWAS that fails to consider the GxG and GxE effects, we extend associative genomics and transcriptomics into a broader associative omics by systematically integrating all available omic data into one analytical model. Here we propose a new LMM and describe the development of a pipeline for analyzing traits through ome-wide association studies (PATOWAS) to implement the model. The proposed LMM considers not only the additive effects of each biological marker but also the interaction effect of each marker pair. The marker pairs’ interaction effect introduced here corresponds to two-dimensional (2D) association mapping, which is complementary to one-dimensional (1D) association mapping in regular GWAS. Consequently, the proposed model and PATOWAS pipeline are not limited to GWAS for genotype-to-phenotype mapping (G2P); instead, they are capable of performing multiple types of ome-wide association studies, such as transcriptome-wide association studies (TWAS) for transcript-to-phenotype mapping (T2P) and metabolome-wide association studies (MWAS) for metabolite-to-phenotype mapping (M2P).

We submit a rice recombinant inbred line (RIL) dataset with three omics markers and two agronomic traits to PATOWAS for comprehensive analyses of associative omics. The results demonstrate that our proposed LMM and the pipeline PATOWAS can effectively address the GxG effect and the GxE effect, perform multiple-level associative omics in one platform, and innovatively provide a systems biology view into the traits analyzed.

## Results

### Associative omics, PATOWAS, and integrative omics

We aimed to systematically integrate multiple associative omic results to provide more biological insights into the phenotypic traits to be analyzed. We first collected a dataset of 210 rice RILs genotyped with 1619 marker bins, profiled with 22,584 transcripts and 1000 metabolites, and phenotyped with two agronomic traits (Table 1). The phenotypic traits (Supplementary Data 1–2) were yield (YIELD) and (kilo-) thousand grain weight (KGW), and the omic quantitative markers (Supplementary Data 3–5) were bin-based genotype data, Affymetrix RNA microarray-based gene expression data, and mass spectrometry-based profiling of metabolite abundance data. We presumed that expressed transcripts, proteins, and metabolites are prone to vary when subjected to the environments, while the genetic variants are considerably stable. Therefore, compared with genome-wide genotypic data, we further presumed that measured gene expression and metabolite abundance contain both gene and environment information and expect that associative transcriptomics (T2P or TWAS) or metabolomics (M2P or MWAS) could explain more phenotypic variance (Supplementary Fig. 1).

**Table 1:** Summary of phenotypic trait data and omic marker data

Trait data (1D vector)		Omics marker data (2D matrix)		
YIELD	KGW	Binned genotype	Expression gene transcript	Metabolite abundance
210 × 1	210 × 1	1619 × 210	22,584 × 210	1000 × 210

**Fig. 1:**
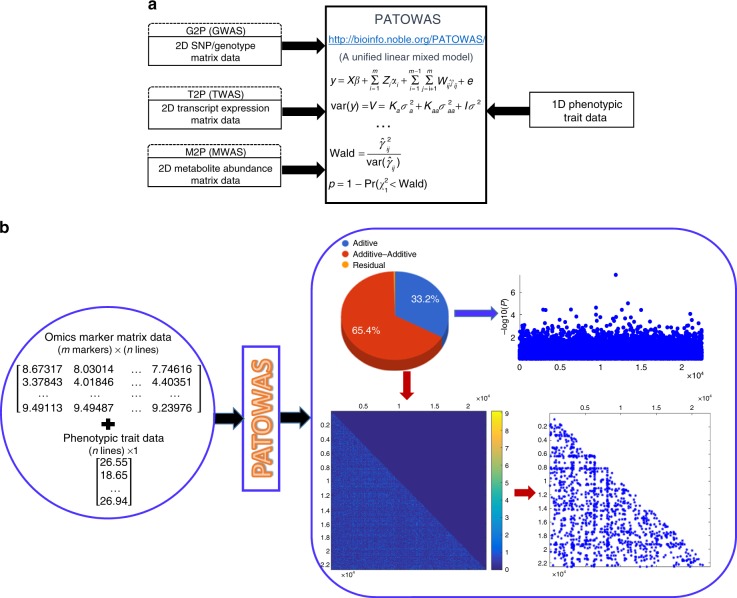
Biological concept of the PATOWAS pipeline and biological insight of an example association mapping resulting from PATOWAS. **a** Three types of omic markers to phenotype association mappings, e.g., (1) genome-wide sequence/genotype variation to phenotypic variation mapping (G2P or GWAS), (2) transcriptome-wide gene expression variation to phenotypic variation mapping (T2P or TWAS), and (3) metabolome-wide metabolite abundance variation to phenotypic variation mapping (M2P or MWAS), can be analyzed using the unified linear mixed model in PATOWAS. **b** PATOWAS needs 2D omics marker matrix data and 1D phenotypic trait data as input. Specific PATOWAS results include (1) variance component analysis result showing two biologically meaningful components: additive, additive–additive, and residual; (2) 1D association mapping for the Additive component; and (3) 2D association mapping for the Additive–Additive component, and further, the significant omics marker pairs extracted by thresholding

Motivated by our consideration of genetic epistasis and our desire to explain more phenotypic variance, we next proposed a statistical LMM that considers not only the additive effects of each marker variant but also the interaction effects of each marker pair. Based on this linear model, we developed a PATOWAS pipeline to analyze traits through multiple ome-wide association studies. Therefore, the proposed model and PATOWAS can be used to study not only GWAS for G2P but also TWAS for T2P and MWAS for M2P, which is progress toward an integrative omics (Fig. 1a).

To test this presumption and verify our consideration, we used PATOWAS to analyze the rice RIL datasets with two agronomic traits and three different omics markers. PATOWAS accepts 2D omics marker matrix data and 1D phenotypic trait data as inputs (Fig. 1b). PATOWAS results for one specific associative omics mainly include three parts: variance component analysis for the partition of phenotypic variance, a 1D association map for the direct biological markers, and a 2D association map for the interaction of biological marker pairs (Fig. 1b). Of the three variance components, the additive component for the markers’ direct effects and the additive–additive component for the marker pairs’ interaction effects are biologically meaningful and can be explained by the linear model. The higher the sum of the two components, the lower the residual component and the more phenotypic variance can be explained by the model. Of all markers’ and marker pairs’ effects, those with higher −log_10_(*p*) values indicate markers or marker pairs that are more relevant to the phenotypic trait.

In the present study, we sequentially submitted three omic marker datasets to PATOWAS to analyze the two field traits, YIELD and KGW. We downloaded the results after completion of the analyses. Based on these results, multiple associative omics and the biological insight can be compared and integrated. For example, the combination of 1D association mapping across G2P and T2P can help identify the genotype and expressed gene transcript markers with consistent physical positions; comparison of the metabolites from 1D M2P association mapping can uncover the biochemical relevance of tissue-specific metabolites and traits to be analyzed; and the investigation of major biomarker pairs from 2D association mapping can be used to build an association network. All these together provide a systems biology view into the analyzed traits, leading toward an answer about how genes, transcripts, proteins, and metabolites work together to produce an observable phenotype.

### Variance component analysis

**Fig. 2:**
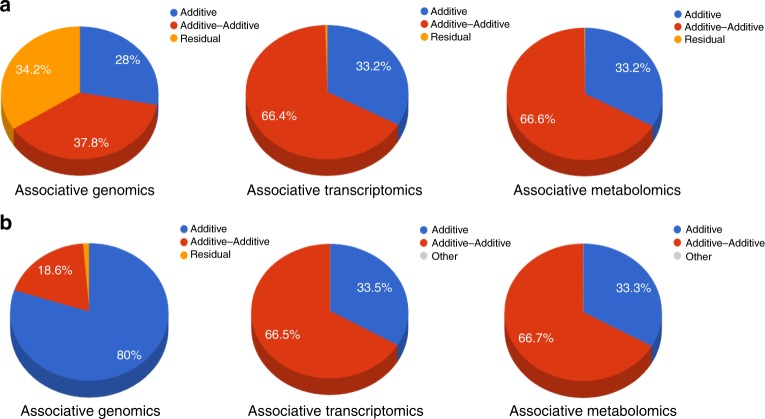
Pie chart illustrations of variance component analysis results for traits YIELD (**a**) and KGW (**b**) across associative genomics, transcriptomics, and metabolomics. Three components in each pie chart are colored with blue, brown, and yellow and represent the three estimated variance ratios of additive, additive–additive, and residual, respectively

Based on the variance component analysis results, we generated six pie charts displaying the three variance components of the two traits across associative genomics, associative transcriptomics, and associative metabolomics (Fig. 2).

We found that the two biologically meaningful variance components accounted for nearly all of the phenotypic trait variance in associative transcriptomics and associative metabolomics but not in associative genomics. Also, YIELD was a more complex trait than KGW, as the two biologically meaningful variance components accounted for only 66% of the total phenotypic variance in associative genomics but nearly 100% of the total phenotypic variance in associative transcriptomics and metabolomics (Fig. 2). These findings demonstrate that a chain of environmentally responsive genes and metabolites can be observed and explained at the transcriptomic and metabolomic levels but not at the genomic level.

Here we noticed that the marker number for transcripts was obviously one-order of scale higher than the other two. Consider the marker-by-marker interactions: The pairwise number of transcripts will reach to ~250 million, which is about two-order of scale larger than the other two kinds of omic markers.

To test whether the higher ratio of biological explanatory components observed in the TWAS result is not due to the larger numbers of transcripts used in TWAS, we further produced a reduced transcript gene set with a number scale comparable to the genotypes and metabolites. We separately submitted the reduced transcript gene set to PATOWAS and checked the variance component analysis result.

The procedures to generate a reduced gene set are described as follows: First we mapped the 22,584 transcript genes into the 1619 genotype bins (Supplementary Data 6); one genotype bin may contain none to hundreds of transcript genes. Based on the 1D association mapping result, at most only one representative transcript in one bin was selected. We chose the transcript with the highest −log_10_(*p*) as the representative transcript of a genotype bin. Then we generated a reduced transcript gene set for each phenotypic trait, which essentially is a data matrix with a dimension of 1543 × 210 (Supplementary Data 7–8). Its number of markers was comparable to those in the analyzed genotypes and metabolites. The same approaches were also used to generate two positional comparable 1D G2P and T2P association mapping results in the following section.

We submitted the reduced transcript data and the two phenotypic traits, KGW and YIELD, to PATOWAS for further study. Based on the variance component analysis results, two additional pie charts displaying the three variance components of the two traits in associative transcriptomics were plotted (Supplementary Fig. 2). Again, we observed that the two biologically meaningful components explained nearly 100% of the phenotypic variance, with only a fluctuation between the two components. Thus, we conclude that the much larger numbers of transcripts used in TWAS is not the reason for the higher explanatory ratio of phenotypic variance in associative transcriptomics.

Our proposed LMM involve two biologically meaningful variance components: $\sigma _{\mathrm {a}}^2$, $\sigma _{\mathrm {aa}}^2$. To measure the portion of phenotypic variance that can be explained by the model, we define the broad-sense heritability by

1 $$H = \frac{{\sigma _{\mathrm {a}}^2 + \sigma _{\mathrm {aa}}^2}}{{\sigma _{\mathrm {a}}^2 + \sigma _{\mathrm {aa}}^2 + \sigma ^2}}$$

Modern GWAS application often involves a panel with hundreds of thousands, or even millions, of genetic variants under only several hundred individual samples^19^. The statistical modeling of such cases is usually challenging because the sample size is substantially smaller than the number of covariates. This is well-known as a “large *p* small *n*” problem^20^ and requires careful assessment of the statistical characteristics^21^.

Our proposed method really can explain more of phenotypic variance, but the cost is that it generates a large number of pairwise covariates. Therefore, it is worthwhile to assess the heritability of the proposed LMM, particularly at the high-dimensional data.

**Fig. 3:**
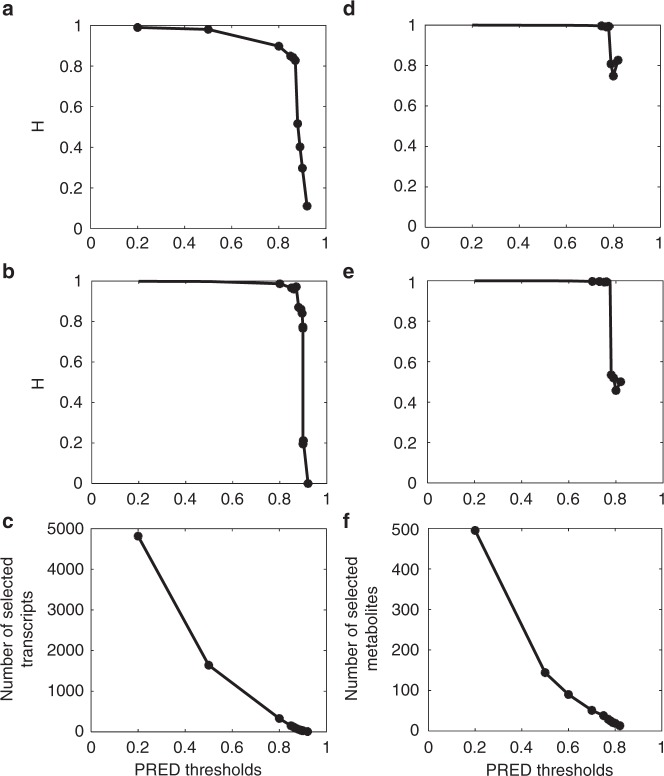
The assessment of broad-sense heritability (*H*) with the selected markers after PRED thresholding. **a** and **b** *H* vs. PRED threshold for trait YIELD and trait KGW in associative transcriptomics. **c** The numbers of selected transcript markers with the applied PRED thresholds. **d** and **e** *H* vs. PRED threshold for trait YIELD and trait KGW in associative metabolomics. **f** The numbers of selected metabolite markers with the applied PRED thresholds

First, the predictability^22^ that is represented by the squared correlation coefficient between the observed and predicted phenotypic value was applied. The squared correlation is approximately equal to *R*^2^ = 1−PRESS/SS, where PRESS is the predicted residual error sum of squares and SS is the total sum of squares of the phenotypic values. In principle, we treated each transcript or metabolite marker as an intermediate phenotypic trait and predicated all of these intermediated phenotypic values from all the genotypic data. Therefore, each transcript or metabolite will have an *R*^2^ value, predictability (PRED). We then used the HAT method^23^ to calculate the PREDs for all transcripts and metabolites (Supplementary Data 9–10), applied a series of variable thresholds to the PREDs, and selected the transcript and metabolite markers. Finally, we submitted the subsets of selected transcript genes and metabolites to PATOWAS for variance component analysis and calculated the broad-sense heritability, *H*. Figure 3 shows the assessment result of the broad-sense heritability (*H*) with the selected markers by PRED thresholding. We found that the number of selected markers continued decreasing as the PRED threshold increased; however, the broad-sense *H* provides us with a very different perspective of different traits and different associative omics. It needs only ~1000 and fewer than 100 transcripts to explain more than 97% of the phenotypic variance in traits YIELD and KGW, respectively. In associative metabolomics, only 30 metabolites are enough to explain more than 90% of the phenotypic variance. In general, trait KGW is more conserved than trait YIELD, and associative metabolomics is more conserved than associative transcriptomics.

Variance component analysis provides us with a big picture by partitioning the phenotypic variation into three components. The two biologically meaningful components for individual markers’ direct effects and the marker pairs’ interaction effects can be further illustrated by 1D and 2D association mapping, respectively.

### 1D association mapping

1D association mappings from PATOWAS across different associative omics can be combined, integrated, and compared, providing biological insights in trait analysis on both system and molecular biology levels.

### Consistency of 1D G2P mapping using PATOWAS and other GWAS tools

Conventional GWAS tools such as TASSEL^24^, GCTA^1^, and PLINK^25^ can build associations between genotypes and phenotypes by calculating and outputting a *p-*value or −log_10_(*p*) value for each genotypic marker. The linear model adopted usually considers only the marker’s direct effect, which is mostly additive. This process essentially is 1D association mapping. PATOWAS is based on our proposed LMM, which considers not only the additive effect for each marker but also the additive × additive interaction effect for each marker pair. Therefore, PATOWAS calculates and outputs a *p-*value for each marker and a *p-*value for each marker pair, which essentially provides both 1D and 2D association mapping.

Regarding G2P mapping, 1D association mapping using PATOWAS can be compared with other GWAS tools^26^. We submitted the same RIL rice genotype and two phenotypic trait data to PATOWAS and TASSEL. We compared the 1D *p-*values returned from both tools and found that the results are very consistent. The Manhattan and Q–Q plots using the same genotype and phenotypic trait data from PATOWAS and TASSEL are illustrated in Supplementary Fig. 3.

### Positional alignment and molecular validation across 1D G2P and T2P mapping

Harper et al.^2^ developed an associative transcriptomic approach to analyzing traits of the polyploid crop *B. napus*. Their method combines SNP-based and gene expression-based association results to identify high-confidence transcription factor candidates. As mentioned before, the 1D *p-*values returned from PATOWAS correspond to the additive effects for the individual markers, and can be used to generate a 1D Manhattan plot. To generate comparable plots between associated genotypic markers and transcript gene markers along their chromosomal position, we first mapped transcript genes to genotype bins and then selected the minimum *p-*value as the representative *p-*value of a bin (Supplementary Data 6). This mapping process between genotype bins and transcript genes ensured that there would be 1619 *p*-values for the two associative omic markers, making it possible to generate aligned 1D plots of −log_10_(*p*) values along the markers’ chromosomal positions.

**Fig. 4:**
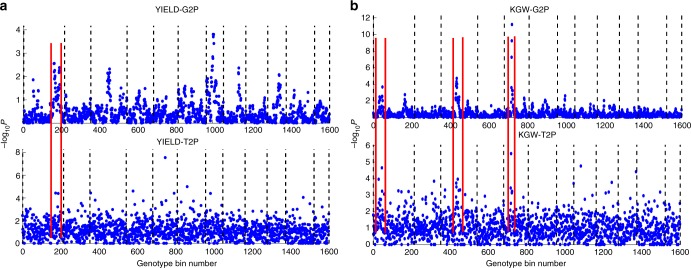
Illustration of the chromosomal position consistency between genomics and transcriptomic markers. **a** Aligning the 1D G2P and T2P association mapping for trait YIELD. **b** Aligning the 1D G2P and T2P association mapping for trait KGW. The maximum −log_10_(*p*) value among the multiple expressed transcript genes in a genotype bin was selected as the representative −log_10_(*p*) value. Dashed lines distinguish the 12 chromosomes and corresponding marker/bin numbers for the complete rice genome. Chromosomal position consistency between G2P and T2P is presented, and the matched local maximum regions are bounded with two red lines

We could easily find the positional consistency between genotype and expressed gene markers (Fig. 4). For YIELD, there was one local maximum region matched between G2P and T2P located in chromosome 1 and bounded with two red lines (Fig. 4a). By contrast, for KGW, most local maximum regions were matched between G2P and T2P (Fig. 4b). Therefore, as KGW is a more specific trait that is less affected by external environmental factors than YIELD, its high genotype variation regions always correspond to high gene expression variation regions pinpointed with high −log_10_(*p*) values in both G2P and T2P.

According to the −log_10_(*p*) values, we focused on trait YIELD and picked up the top 10 transcript gene markers for a deep molecular function investigation. The top 10 transcript gene markers are distinguished with a unique index and can be identified by its gene locus ID. Through a literature search, we found that at least five of the top 10 transcript markers have been reported to biologically affect rice YIELD (Supplementary Table 1). For example, marker T_2925 (*LOC_Os01g62860*) was reported to be related to seed shattering^27^; marker T_3229 (*LOC_Os01g67580*) was reported to be related to drug resistance^28^; and markers T_6368 (*LOC_Os03g03070*) and T_13429 (*LOC_Os06g11330*) were reported to control or delay flowering time^29,30^. Marker T_11921 (*LOC_Os05g31040*) in particular acquired the highest significance value (−log_10_(*p*) = 7.53) and was reported as the CKX9 plant hormone gene that could lead to the accumulation of cytokinin and the increased tiller number^31,32^. All these literature-validated gene markers demonstrate that our PATOWAS has the capability to perform trusted association mapping between causal expressed transcript variants and the resulting phenotypes. We annotated and marked these five genes to the 1D T2P association mapping plot and found that most of them belong to high association peaks (Supplementary Fig. 4). The aims of associative genomics or transcriptomics are to find the genetic variants or expressed transcript variant, which can obviously affect the phenotypic trait. Therefore, the high genotype variation or high gene expression variation regions warrant further study. For YIELD, there is only one obvious consistent matched region between G2P and T2P, and it falls into the surroundings of markers T_2925 and T_3229. However, other transcript gene markers, such as T_11921 with its highest significance value of 7.5309, do not fall into the high genotype variation region (Supplementary Fig. 4, Region C). Therefore, we could conclude that PATOWAS and the associative transcriptomics capture not only the inheritable genetic information from the genome but also the intermediated environmental information at the transcriptome level.

### 1D M2P association mapping and comparison of metabolite markers between leaf and seed

**Fig. 5:**
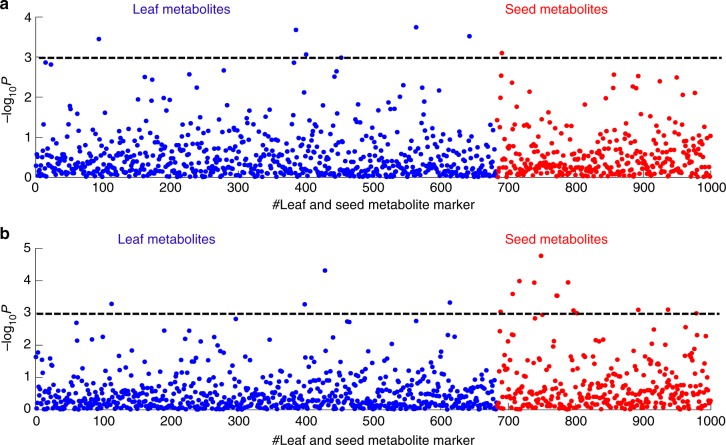
Illustration of the 1D M2P association mapping results. Scatter plot of the 1D M2P association mapping for traits YIELD (**a**) and KGW (**b**). The M2P analysis in this study includes 683 leaf metabolites and 317 seed metabolites, and the two kinds of metabolites are colored with blue and red in the two scatter plots. A tentative significance threshold bar (−log_10_(*p*) = 3.0) is set up to show the relevance of the metabolite markers with the phenotypic trait

In the present study, we used PATOWAS to analyze the association of 1000 metabolites with traits YIELD and KGW and then plotted the 1D M2P association results across individual metabolites (Fig. 5).

In genetic association analysis, determining the correct *p-value* threshold is always critical and subjective^33^. To tell a methodology story, we tentatively set the threshold as *p* = 0.001, and the metabolite marker could be considered significant if $- {\mathrm {log}}_{10}(p) \ge 3.0$. Obviously, we could observe that there were more significant (−log_10_(*p*) ≥ 3.0) metabolite markers from leaf than from seed for YIELD (Fig. 5a), whereas there were more significant (−log_10_(*p*) ≥ 3.0) metabolite markers from seed than from leaf for KGW (Fig. 5b).

Further, we picked up variable top *n* significant metabolite markers from the total 1000 metabolites and classified them as metabolites from leaf and seed. Table 2 gives the relationship of the variable top *n* with the number of significant (−log_10_(*p*) ≥ Significance_Th) leaf and seed metabolites. Considering that there are 683 and 317 metabolites from leaf and seed, respectively, we set 0.683 and 0.317 as two meaningful ratio thresholds for significant metabolites from leaf and seed. From Table 2, we found that (1) for YIELD, when top *n* < 25, significant leaf metabolites against total top *n* metabolites always have a ratio higher than 0.683; and (2) for KGW, when top *n* < 500, the significant seed metabolites against the total top *n* metabolites usually produce a ratio higher than 0.317.

**Table 2:** Summary of variable top *n* significant (−log_10_(*p*) ≥ Significance_Th) metabolites from leaf and seed across two traits

Top#	YIELD			KGW		
	Significance_Th	No. of leaf metabolites	No. of seed Metabolites	Significance_Th	No. of leaf metabolites	No. of seed metabolites
5	3.0904	4	1	3.9283	1	4
10	2.8044	9	1	3.2709	3	7
15	2.5274	12	3	3.0274	4	11
20	2.4269	15	5	2.8044	5	15
25	2.2298	17	8	2.5503	9	16
30	2.1278	19	11	2.3214	11	19
50	1.6936	33	17	2.0310	22	28
100	1.2402	64	36	1.4739	49	51
200	0.8616	134	66	0.9114	110	90
500	0.3310	333	167	0.3669	312	188

All these results suggest that leaf metabolites are more relevant to YIELD, while seed metabolites are more relevant to KGW, which is consistent with the findings of Xu et al.^34^. This could be explained by the fact that the photosynthesis process takes place mainly in leaf tissue and is the main factor determining rice yield^35^.

Further, we focused on the top 10 significant metabolites for deep molecular function investigation. Based on a literature search, the identification and classification of the top 10 metabolites are summarized in Supplementary Table 2. Of the 10 metabolites, five were identified and two were further classified as flavonoid, of which content was reported as an assessment of the crop yield^36^.

### 2D association mapping

The biological interpretation of 2D association mapping for marker pairs’ interaction effect can be illustrated by visualizing the 2D association matrix directly, significance thresholding, and constructing weighted association networks, etc.

### Illustration of marker pairs’ interaction effect and its significance thresholding

**Fig. 6:**
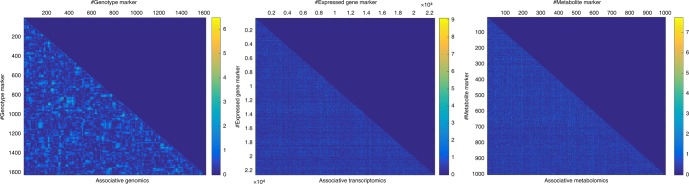
The pseudo-color images that illustrate the 2D association mapping results, representing the marker pairs’ interaction effects for the trait YIELD across associative genomics, associative transcriptomics, and associative metabolomics. The pseudo-color scaling maps the −log_10_(*p*) value from low (deep blue) to high (yellow)

For trait YIELD, three 2D association mapping results were analyzed, and each association matrix was illustrated as a scaled image with pseudocolor (Fig. 6). By comparison, we found that genotypic markers were neighbor-dependent, as evidenced by the clustering of dots, whereas expressed transcript gene and metabolite markers were neighbor-independent, as evidenced by a random distribution of dots. This phenomenon could be explained by the existence of linkage disequilibrium (LD) blocks in population genetics^37^.

We are usually interested in the significant (−log_10_(*p*) ≥ Significance_Th) marker pairs instead of all the marker pairs. Similar to 1D association mapping, we could set a significance threshold to generate a binarized version of the 2D association matrix (Supplementary Fig. 5). We further zoomed in to a specified local region for each associative omics and found that associative genomics demonstrated a 2D local rectangular array while the associative transcriptomics and associative metabolomics showed a 1D local strip (Supplementary Fig. 5 inset). The specificity of the 2D local structure pattern for associative genomics was due to the existence of LD blocks in genomics level. Further, the dimension size of 2D local rectangular array corresponds to the LD block size.

### Conditional 1D association mapping and weighted association network

To a specific omics marker pair, say, markers *X* and *Y*, there is a *p*(*X*, *Y*) value and its significance measured by −log_10_(*p*(*X*, *Y*)), which shows how much the omics marker pair is relevant to the phenotypic trait to be studied. If we pinpoint a marker pair (*X*, *Y*) to the image illustrated for 2D association mapping, there surely are two specific lines recorded by 1D association significance values (Supplementary Fig. 6). To each associative omics, we selected a representative marker pair, and for each representative marker pair, we marked the two specific lines as white and red and generated two corresponding conditional 1D association mapping plots (Supplementary Fig. 6, middle and bottom). Here, the conditional 1D association mapping originally came from the 2D association matrix, which biologically means how much the other omics markers interconnected with the selected marker to affect the studied phenotypic trait. We found that the conditional 1D associative genomics mapping could provide us with some obvious QTLs, while the conditional 1D associative transcriptomics and metabolomics mapping showed us random association mapping.

Further, if we focus on one specific omics marker and set a significance threshold, its interactive pairs along the vertical or horizontal axes with higher −log_10_(*p*) values can be considered relevant regulators of that specific marker. Then an association network centered on the specific omics marker could be constructed. The tie connecting two omics marker nodes has an assigned association significance values. This could be called a weighted association network, which is very different from the co-expression-based gene regulation network^38^ due to it having a direct biological meaning with the phenotypic trait to be studied.

According to the marker pairs’ significance values, we picked up top 10 associative transcript and metabolite marker pairs. To acquire a deep molecular-level investigation, we conducted a comprehensive literature search and function annotation for two types of associative omics marker pairs (Supplementary Tables 3–4).

**Fig. 7:**
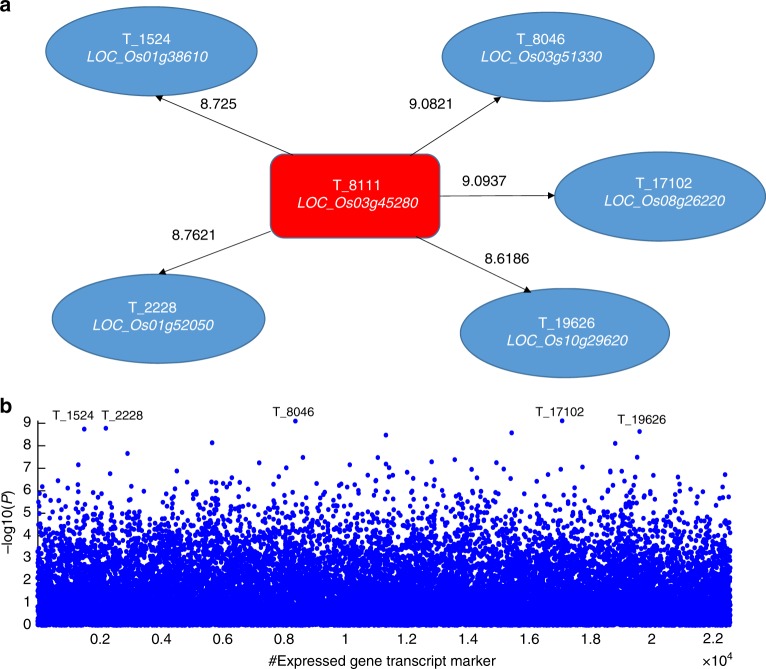
A weighted expressed gene marker interaction network. **a** Illustration of the association network. Five of the top 10 expressed gene transcript marker pairs are interconnected with a hub transcript T_8111 (*LOC_Os03g45280*) and used to construct an example association network. The weight on each tie is the significance value measured by −log_10_(*p*). **b** Plot of conditional 1D association mapping for transcript marker T_8111. For the transcript T_8111 (*LOC_Os03g45280*), its conditional 1D association mapping was extracted from the 2D association matrix

Of the top 10 transcript marker pairs, most of the expressed transcripts are molecularly functional relevant to plant growth, plant hormones, cold and drought stress, etc. (Supplementary Table 3), which can finally affect the phenotypic trait YIELD. In addition, five transcript marker pairs are interconnected with one hub transcript T_8111(*LOC_Os03g45280*). Therefore, a hub transcript T_8111 (*LOC_Os03g45280*)-centered expressed gene association network has been tentatively constructed (Fig. 7).

Although the current knowledge about metabolite identification is very limited, we found that most of the identified metabolites from the top 10 metabolite marker pairs were classified into flavonoid or phenolic (Supplementary Table 4). There have been reports that the total phenolic and flavonoid content was comparably relevant to the crop final product yield^36^. Of the top 10 association metabolite marker pairs, we found four marker pairs were centered on unknown metabolite marker M_195 and two marker pairs were centered on flavonoid metabolite marker M_311 (Supplementary Fig. 7).

## Discussion

We extended the concept of genome-wide association to a broader concept of ome-wide association. To overcome the limitations of regular additive GWAS models that fail to consider epistatic and environmental interaction effects, we proposed a new LMM and successfully developed a new PATOWAS pipeline for ome-wide association studies.

We presume that the measured data of gene expression in the transcriptome and metabolite abundance in the metabolome contain not only heritable, stable genetic information but also fluctuating environmental information. Thus, the systematic integration and analysis of multiple levels of associative omics data can provide panoramic insight for complex trait analysis.

To test and validate our presumption, we analyzed a dataset of 210 RILs of rice consisting of genomic, transcriptomic, and metabolomic markers as well as two agronomic phenotypic traits using PATOWAS. The results of the three associative omics analyses were integrated and compared to perform complete trait analysis.

Compared with the genotype-based G2P association, the variance component analysis of gene expression-based T2P and metabolite abundance-based M2P association explained nearly 100% of the phenotypic variance, supporting our presumption that measured gene expression and metabolite abundance data contain both gene and environment information. For KGW, genotype-based G2P association explained >98% of the phenotypic variance, suggesting that this is a simple trait that is less affected by the environment. Of the two types of biologically meaningful variance components, the additive component corresponding to individual genetic markers accounted for 80% of the phenotypic variance, further suggesting that KGW is a more heritable trait that can be easily manipulated by breeding. However, for YIELD, genotype-based G2P association explained only 66% of the phenotypic variance, suggesting that it is a more complex trait that is easily affected by the external environment. In addition, the additive genetic component accounted for only 28% of the phenotypic variance, suggesting that YIELD is more difficult to manipulate by breeding. However, as YIELD may be the most important agronomic trait, environmental factors that affect the transcriptome and metabolome should be carefully considered to produce improvements in this trait.

We found obvious consistencies in genome coordinates between associated genotype markers and expressed gene transcript markers, allowing us to identify high-confidence, co-verified genotype and transcript markers for the same trait and suggesting the presence of correlations between SNP-based genotype data and transcriptomic data. Compared with trait YIELD, trait KGW had more consistent regions between G2P and T2P, indicating that more correlated information was transferred from the genomic level to the transcriptomic level.

We also investigated inconsistent regions between the G2P and T2P plots for YIELD (Supplementary Fig. 4). On chromosome 3, there was a local maximum peak in the G2P plot but not in the T2P plot (Supplementary Fig. 4, Region B), whereas on chromosome 5, there was a local maximum peak in the T2P plot but not in the G2P plot (Supplementary Fig. 4, Region C). We have verified that the latter was the CKX9 plant hormone gene, which could lead to the accumulation of cytokinin and affect the rice grain yield^31,32^. We speculate that these inconsistencies occurred because of environmental fluctuations resulting in the downregulation of genes located in Region B, and the upregulation of the CKX9 gene located in Region C.

Our associative metabolomic results from PATOWAS indicated that there were more leaf metabolites than seed metabolites relevant to YIELD and vice versa for KGW, suggesting that significantly associated metabolites are tissue-specific and trait-specific. In contrast to Xu et al.’s method ^34^, which provides only global information, our PATOWAS results provide details about how relevant each metabolite is to YIELD and KGW.

Although there are more than 200,000 different metabolites in the plant kingdom^39^, only a few hundred have been able to be measured in one experiment. Furthermore, because of technical bottlenecks in metabolite identification, most measured metabolites are unannotated^40^. If we can increase the number of measured and identified metabolites, the metabolome-wide association results from PATOWAS will become more accurate. Furthermore, if we can link associated genes with known metabolites, we can find and explain new pathways connecting enzymatic genes with their eventual metabolites.

The 2D *p-*value scanning results from PATOWAS can be used to construct an association network. Such an association network is trait-related and also can be constructed and analyzed for different ome-wide association studies. The integration of multiple layers of ome-wide association networks, together with other results of PATOWAS analysis, can provide panoramic biological insight for trait analysis, leading toward an answer to the question of how genes, transcripts, proteins, and metabolites work together to produce an observable phenotype.

## Methods

### Statistical method

*A new LMM incorporating additive and interaction effects*: We proposed a new LMM for multiple associative omics, mathematically described below, that incorporates all markers’ direct additive effects and marker pairs’ interaction effects.

Let *y* be an *n* × 1 vector of a quantitative phenotypic trait and *Z* be an *m* × *n* marker matrix for a quantitative omic dataset, such as coded genotypic data, transcript gene expression data, or metabolite abundance data. Coded genotypic data can be acquired by sequencing and genotyping a population^41^, gene expression data can be acquired by microarray hybridization or mRNA-seq experiments, and metabolite abundance data can be acquired by gas chromatography–mass spectrometry or liquid chromatography–mass spectrometry followed by metabolite feature extraction, annotation, alignment, and quantification^42–44^.

The LMM that incorporates the markers’ additive effects and marker pairs’ interaction effects can be represented as

2 $$y = X\beta + \mathop {\sum}\limits_{{\it{i}}{\mathrm{ = 1}}}^m {Z_ia_i} + \mathop {\sum}\limits_{i = 1}^{m - 1} {\mathop {\sum}\limits_{j = i + 1}^m {W_{ij}\gamma _{ij}} + e}$$

where *X* is an *n* × 1 vector of unity and *β* is the intercept; *Z*_*i*_is the *i*th column of matrix *Z*, and *a*_*i*_ is the *i*th marker’s additive effect on the trait; $W_{ij} = Z_i \ast Z_j$ is the element-wise product of vectors *Z*_*i*_ and *Z*_*j*_; *γ*_*ij*_ is the interaction effect between marker *i* and marker*j*; and *e* is an *n* × 1 vector of residual error.

We treat each marker’s effect as a randomly distributed normal variable with a mean of zero and a common variance across all markers or pairs of markers, as shown by $a_i \sim N(0,\sigma _{\mathrm {a}}^2)$ and $\gamma _{ij}\sim N(0,\sigma _{\mathrm {aa}}^2)$. The residual errors are of $e\sim N(0,\sigma ^2)$. The total additive and interaction effects are denoted by: $\mathop {\sum}\limits_{i = 1}^m {Z_ia_i}$ and $\mathop {\sum}\limits_{i = 1}^{m - 1} {\mathop {\sum}\limits_{j = i + 1}^m {W_{ij}\gamma _{ij}} }$, respectively.

The expectation of the model is *E*(*y*) = *Xβ*, and the variance is

3 $${\mathrm{var}}\left( y \right) = K_{\mathrm {a}}\sigma _{\mathrm {a}}^2 + K_{\mathrm {aa}}\sigma _{\mathrm {aa}}^2 + I\sigma ^2$$

where *K*_a_ and *K*_aa_ are marker-generated additive and epistatic kinship matrices with values calculated by formulas (4) and (5).

4 $$\begin{array}{l}K_{\mathrm {a}} = \frac{1}{d_{\mathrm {a}}}\mathop {\sum}\limits_{i = 1}^m {Z_i} Z_j^T \hfill \\ K_{\mathrm{aa}} = \frac{1}{{d_{\mathrm{aa}}}}\mathop {\sum}\limits_{i = 1}^{m - 1} {\mathop {\sum}\limits_{j = i + 1}^m {W_{ij}W_{ij}^T} \hfill } \end{array}$$

where

5 $$\begin{array}{l}d_{\mathrm {a}} = \frac{1}{n}tr\left( {\mathop {\sum}\limits_{i = 1}^m {Z_iZ_j^T} } \right)\hfill \\ d_{\mathrm {aa}} = \frac{1}{n}tr\left( {\mathop {\sum}\limits_{i = 1}^{m - 1} {\mathop {\sum}\limits_{j = i + 1}^m {W_{ij}W_{ij}^T} } } \right)\end{array}$$

are normalization factors that allow the *K* matrices to have diagonal elements as close to unity as possible.

The model involves three variance components, $\sigma _{\mathrm {a}}^2$, $\sigma _{\mathrm {aa}}^2$, and *σ*^2^, which can be estimated by the restricted maximum likelihood (REML) method for dissection of phenotypic variance.

### Estimating variance components using the REML method

The model to estimate variance component is

6 $$y = X\beta + \xi + \zeta + e$$

where ξ and ζ are the additive and interaction effects, respectively. The expectation of the model is *E*(*y*) = *Xβ*, and the variance is

7 $${\mathrm {var}}\left( y \right) = {\mathrm {var}}\left( \xi \right) + {\mathrm {var}}\left( \zeta \right) + {\mathrm {var}}\left( e \right) = K_{\mathrm {a}}\sigma _{\mathrm {a}}^2 + K_{\mathrm {aa}}\sigma _{\mathrm {aa}}^2 + I\sigma ^2$$

The restricted log-likelihood function is

8 $$L(\beta ,\sigma _{\mathrm {a}}^2,\sigma _{\mathrm {aa}}^2,\sigma ^2) = - \frac{1}{2}\ln \left| V \right| - \frac{1}{2}\ln \left| {X^TV^{ - 1}X} \right| - \frac{1}{2}(y - X\beta )^TV^{ - 1}(y - X\beta )$$

Given $\sigma _{\mathrm {a}}^2$, $\sigma _{\mathrm {aa}}^2$, and *σ*^2^, we can solve for *β* by

9 $$\hat \beta = (X^TH^{ - 1}X)^{ - 1}X^TH^{ - 1}y$$

Substituting Eq. (9) into Eq. (8) gives

10 $$\begin{array}{l}L(\sigma _{\mathrm {a}}^2,\sigma _{\mathrm {aa}}^2,\sigma ^2) = - \frac{1}{2}\ln \left| H \right| - \frac{1}{2}\ln \left| {X^TH^{ - 1}X} \right| - \frac{1}{{2\sigma ^2}}(y - X\beta )^TH^{ - 1}(y - X\beta ) \\ \qquad \qquad \qquad + \frac{{n - r(X)}}{2}\ln (\sigma ^2)\hfill\end{array}$$

Therefore, the defined likelihood function has three unknowns. Calling any optimization subroutine, we can obtain the REML estimates of the three variance components.

After the three variance components are acquired, we fix the variance ratio $\hat {\lambda}_{\mathrm{a}} = \hat {\sigma}_{\mathrm {a}}^{2}/\hat {\sigma} ^2$, $\hat {\lambda} _{\mathrm {aa}} = \hat {\sigma} _{\mathrm {aa}}^{2}{/}\hat {\sigma}^2$ and estimate and test the additive effects and interaction effects by conducting 1D scanning across all markers and 2D scanning across all marker pairs, respectively.

### 1D and 2D scanning to estimate additive and interaction effects

We define model I and use it to estimate the additive effect of marker *Z*_*i*_ as shown below:

11 $$y = X\beta + Z_ia_i + e$$

The expectation of this model is

12 $$E(y) = X\beta + Z_ia_i$$

We also define model II and use it to estimate the interaction effect of marker pair *W*_*ij*_ as shown below:

13 $$y = X\beta + Z_ia_i + Z_ja_j + W_{ij}\gamma _{ij} + e$$

The expectation of this model is

14 $$E(y) = X\beta + Z_ia_i + Z_ja_j + W_{ij}\gamma _{ij}$$

When (*λ*_a_,*λ*_aa_) are fixed, the two models are fixed models and can be solved using the weighted least-squares method. The variance of each model can be written as

15 $$\begin{array}{ccccc}\\ V = {\mathop{\rm{var}}} (y) &=& K_{\mathrm {a}}\sigma _{\mathrm {a}}^2 + K_{\mathrm {aa}}^{}\sigma _{\mathrm {aa}}^2 + I\sigma ^2\\ &=& \left( {K_{\mathrm {a}}\lambda _{\mathrm {a}} + K_{\mathrm {aa}}\lambda _{\mathrm {aa}} + I} \right)\sigma ^2 \\ &=& \left( {H + I} \right)\sigma ^2\hfill\\ \end{array}$$

Furthermore, we define a parameter matrix *P* and a vector *b* as the following:

16 $$P = \left\{ {\begin{array}{*{20}{c}} {\left[ {X||Z_i} \right]} & {{{{\rm{Model}}}\quad {\mathrm{I}}}} \\ {\left[ {X||Z_i||Z_j||W_{ij}} \right]} & {{{{\rm{Model}}}\quad {\mathrm{II}}}} \end{array}} \right.$$

17 $$b = \left\{ {\begin{array}{*{20}{c}} {\left[ {\beta //a_i} \right]} & {{{{\rm{Model}}}\quad {\mathrm{I}}}} \\ {\left[ {\beta //a_i//a_j//\gamma _{ij}} \right]} & {{{{\rm{Model}}}\quad {\mathrm {II}}}} \end{array}} \right.$$

where *P* is a *n* × 2 or *n* × 4 matrix that concatenates all matrices horizontally and *b* is a 2 × 1 or 4 × 1 vector that concatenates all regression coefficients vertically. The generalized least square estimate of *b* is

18 $$\hat b = (P^TV^{ - 1}P)^{ - 1}P^TV^{ - 1}y$$

Note that

19 $$V^{ - 1} = (H + I)^{ - 1}{\mathrm{/}}\sigma ^2$$

Therefore,

20 $$\hat b = (P^T(H + I)^{ - 1}P)^{ - 1}P^T(H + I)^{ - 1}y$$

Note that when (*λ*_a_,*λ*_aa_) are fixed, the following matrix is a constant matrix and can be simplified using Eigen decomposition:

21 $$(H + I)^{ - 1} = (UDU^T + I)^{ - 1} = U(D + I)^{ - 1}U^T$$

where *D* (a diagonal matrix) holds the eigenvalues of *H*, and *U* (a matrix) holds the eigenvectors of matrix *H*. The inverse of *D* + *I* is simply

22 $$(D + I)^{ - 1} = {\mathrm {diag}}\left\{ {\frac{1}{{\delta _i + 1}}} \right\}$$

Rewriting Eq. (20) gives

23 $$\hat b = (P^T(H + I)^{ - 1}P)^{ - 1}P^T(H + I)^{ - 1}y = (P^{ \ast T}WP^ \ast )^{ - 1}P^{ \ast T}Wy^ \ast$$

where

24 $$\left\{ {\begin{array}{*{20}{l}} {P^ \ast = U^TP} \hfill \\ {y^ \ast = U^Ty} \hfill \\ {W = (D + I)^{ - 1}} \hfill \end{array}} \right.$$

The residual error variance is estimated using

25 $$\hat \sigma ^2 = \frac{1}{{n - r(P)}} = (y^ \ast - P^ \ast \hat b)^TW(y^ \ast - P^ \ast \hat b)$$

where *r*(*P*) = 2 and *r*(*P*) = 4 for model I and model II, respectively. The variance matrix of the estimated effects is

26 $${\mathop{\rm{var}}} (\hat b) = (P^{ \ast T}WP)^{ - 1}\hat \sigma ^2$$

For model I,

27 $${\mathop{\rm{var}}} (\hat b) = \left[ {\begin{array}{*{20}{c}} {{\mathop{\rm{var}}} (\hat \beta )} & {{\mathop{\rm{cov}}} (\hat \beta ,\hat a_i)} \\ {{\mathop{\rm{cov}}} (\hat a_i,\hat \beta )} & {{\mathop{\rm{var}}} (\hat a_i)} \end{array}} \right]$$

and the Wald test for H_0_: *a*_*i*_ = 0 is

28 $${\mathrm {Wald}} = \frac{{\hat a_i^2}}{{{\mathop{\rm{var}}} (\hat a_i)}}$$

For model II,

29 $${\mathop{\rm{var}}} (\hat b) = \left[ {\begin{array}{*{20}{c}} {{\mathop{\rm{var}}} (\hat \beta )} & {{\mathop{\rm{cov}}} (\hat \beta ,\hat a_i)} & {{\mathop{\rm{cov}}} (\hat \beta ,\hat a_j)} & {{\mathop{\rm{cov}}} (\hat \beta ,\hat \gamma _{ij})} \\ {{\mathop{\rm{cov}}} (\hat a_i,\hat \beta )} & {{\mathop{\rm{var}}} (\hat a_i)} & {{\mathop{\rm{cov}}} (\hat a_i,\hat a_j)} & {{\mathop{\rm{cov}}} (\hat a_i,\hat \gamma _{ij})} \\ {{\mathop{\rm{cov}}} (\hat a_j,\hat \beta )} & {{\mathop{\rm{cov}}} (\hat a_j,\hat a_i)} & {{\mathop{\rm{var}}} (\hat a_j)} & {{\mathop{\rm{cov}}} (\hat a_j,\hat \gamma _{ij})} \\ {{\mathop{\rm{cov}}} (\hat \gamma _{ij},\hat \beta )} & {{\mathop{\rm{cov}}} (\hat \gamma _{ij},\hat a_i)} & {{\mathop{\rm{cov}}} (\hat \gamma _{ij},\hat a_j)} & {{\mathop{\rm{var}}} (\hat \gamma _{ij})} \end{array}} \right]$$

and the Wald test for H_0_: *γ*_*ij*_ = 0 is

30 $${\mathrm {Wald}} = \frac{{\hat \gamma _{ij}^2}}{{{\mathop{\rm{var}}} (\hat \gamma _{ij})}}$$

The *p-*value for a marker’s additive effect or the interaction effect of a marker pair is calculated using

31 $$p = 1 - \Pr (\chi _{1}^{2} < {\mathrm {Wald}})$$

### PATOWAS pipeline

**Fig. 8:**
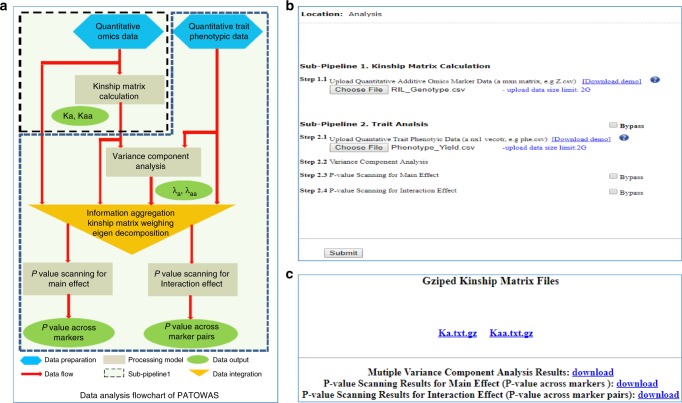
PATOWAS data analysis flowchart and the user interfaces. **a** PATOWAS data analysis flowchart. PATOWAS includes four processing models, need omics marker matrix data and phenotypic trait data as input, and output results kinship matrix, variance component ratios, 1D and 2D *p-*values for main and interaction effects. **b** User interface for submitting data. **c** User interface for downloading results

PATOWAS was developed for analyzing traits through ome-wide association studies. The PATOWAS is composed of two primary sub-pipelines. Sub-pipeline 1 consists of one module designed for kinship matrix calculation, and sub-pipeline 2 is designed for association mapping and integrates three related analysis modules: one for the three variance component analysis, another for 1D *p*-value scanning for all markers’ direct additive effects, and a third for 2D *p*-value scanning for all marker pairs’ interaction effects. The four modules are designated km_cal, vc_anal, ps_main, and ps_inter, respectively. The modules were coded with C/C++ using Code::Blocks in a Linux environment and compiled into four separate executable commands. Several Perl and Linux C shell scripts were developed to function as a wrapper to streamline the complete analysis pipeline. Briefly, when the coded genotype data, transcript gene expression data, or metabolite abundance data are provided, module km_cal calculates and delivers the corresponding kinship matrix. When phenotypic quantitative trait data are provided, module vc_anal estimates and delivers the three variance component ratios utilizing both the quantitative trait data and the available kinship matrices. After performing various information aggregation procedures, including kinship matrix weighing and matrix eigen-decomposition, modules ps_main and ps_inter calculate and return 1D *p*-values for all markers and 2D *p*-values for all marker pairs, respectively (Fig. 8a).

PATOWAS accepts 2D omic marker matrix data and 1D phenotypic trait data in.csv format as inputs (Fig. 8b). After data submission, PATOWAS calculates kinship matrix *K*_a_, *K*_aa_ and uses the intermediate kinship matrix and phenotypic trait data to estimate the variance component ratio *λ*_a_, *λ*_aa_. Finally, a Wald test is employed to scan the 1D and 2D *p*-values for the markers’ additive effects and the interaction effects of marker pairs, respectively (Fig. 8a). The PATOWAS analysis output includes the kinship matrix, estimated variance components, and 1D and 2D *p*-values for the markers’ additive effects and marker pairs’ interaction effects (Fig. 8c). Based on the results, the three variance components accounting for phenotypic variance and the −log_10_(*p*) values for the markers’ additive effects and marker pairs’ interaction effects can be visualized and further analyzed.

To increase the flexibility of analyses, users are allowed to run only a portion of the PATOWAS pipeline according to the input data and user-configured parameters (e.g., users can perform only kinship matrix calculations and the three variance component analyses or only kinship matrix calculations). Such configuration flexibility enables users to utilize PATOWAS to generate specific data, such as a kinship matrix, for their own genetic data analysis.

Similar to PEPIS^8^, PATOWAS was implemented in C/C++ programming language. Furthermore, its computationally demanding analysis modules were implemented using parallel computing techniques, which effectively divide large computational tasks into smaller jobs that are distributed to nodes on networked Linux clusters to accelerate computing.

Let *n* be the number of individuals and *m* be the number of omic markers. The total number of effects is *m* + *C*(*m*,2) = *m*(*m* + 1)/2. According to the kinship matrix calculation formula^7^ and complexity analysis^8^, the multiplications to calculate the kinship matrix *K*_a_ and *K*_aa_ are *mn*(*n* + 1)/2 and *m*(*m*−1)*n*(*n* + 1)/4, respectively. These calculation complexities demonstrate the enormity of the multiplication demand associated with kinship matrix calculations, especially when both *n* and *m* are large. However, the procedure used to calculate each matrix cell value is the same; thus, all *n*(*n* + 1)/2 loops for matrix cell calculation can be parallelized.

The variance component analysis module essentially needs only one optimization for a three-parameter log-likelihood estimation. The 1D additive effects *p-*value scanning module needs *m* Chi-square calculations and Wald tests, and the 2D interaction effects *p-*value scanning module needs *m*(*m*−1)/2 Chi-square calculations and Wald tests. However, the procedure to calculate the *p-*values is the same, so the *m* times additive effect *p-*value calculation and *m*(*m*−1)/2 times interaction effect *p-*value calculation can also be parallelized. The strategy utilized in the PATOWAS for parallel high-performance distributed computing is summarized in Table 3.

**Table 3:** Summary of parallel strategy of PATOWAS for high-performance distributed computing

Computing module	Computation complexity description	Repetitive parallelizable calculation unit	Allocated job for each CPU node with *p* parallelizable nodes
Kinship matrix calculation	$\frac{{n(n + 1)}}{2}$ Loops for each of the 2 kinship matrix’s cell calculations	Kinship matrix cell calculations	$\frac{{n(n + 1)}}{{2p}}$ loops for each matrix’s cell calculations
*p-*Value scanning for main effects	*m* times Wald test and Chi-square calculation	2 degrees of freedom Wald test and Chi-square calculation	$\frac{m}{p}$ times 2 degrees of freedom Wald test and Chi-square calculation
*p-*Value scanning for interaction effects	$\frac{{m(m - 1)}}{2}$ times Wald test and Chi-square calculation	4 degrees of freedom Wald test and Chi-square calculation	$\frac{{m(m - 1)}}{{2p}}$ times 4 degrees of freedom Wald test and Chi-square calculation

### Rice omics data

We analyzed YIELD and KGW from 210 RILs of rice described by Hua et al.^45,46^. The 210 RILs were derived by single-seed descent from a cross between the Zhenshan 97 and Minghui 63 rice hybrids. Field phenotypic data pertaining to YIELD and KGW were collected from replicated field trials on the Huazhong Agricultural University Experimental Farm in Wuhan, China.

Ome-wide quantitative marker data consisted of bin-based genotype data, Affymetrix RNA microarray-based gene expression data, and mass spectrometry-based profiling metabolite abundance data. Over 270,000 high-density SNP markers were used to infer recombination breakpoints (crossovers), which were then used to construct a total of 1619 genotype bins^41^. Each bin was treated as a new synthetic marker for association studies, and the bin map was constructed by genotyping the RIL population sequences.

The transcriptomic data originally consisted of 24,994 expressed genes, which were sampled and measured from flag leaves for all 210 RILs in 2008. Each line had two biological replicates, but RNA extracted from the two replicates was mixed at a 1:1 ratio before microarray expression profiling. The original expression levels were then log_2_-transformed before analysis^47^. Of the 24,994 genes, 22,584 were clearly matched to 1619 genotype bins. We found only minor and inconsequential differences between the analysis results for 24,994 versus 22,584 genes.

The metabolomic data consisted of 683 metabolites measured from flag leaves and 317 metabolites measured from germinated seeds^48^. Metabolomic data were collected in 2009 and 2010. Before mass spectrometry-based metabolic profiling, germinated seeds were sampled in one biological replicate in 2009 and one in 2010, and flag leaves were sampled in two biological replicates in 2009. For both tissues, the abundance level of each metabolite was log_2_-transformed. For each line, we took the average of two replicates’ abundance levels as the measurement of the metabolite.

In summary, the bin genotype data, microarray-based gene expression data, and mass spectrometry-based metabolite data were acquired and stored in three matrices as dimensions of *m* × *n* = 1619 × 210, *m* × *n* = 22,584 × 210, and *m* × *n* = 1000 × 210, respectively (Table 1). Here, *m* and *n* represent the number of markers and individuals, respectively.

### Code availability

The PATOWAS pipeline and source code are freely available at http://bioinfo.noble.org/PATOWAS/. In addition, the source code of PATOWAS has been deposited into the public repository GitHub at https://github.com/ZhaoBioinformaticsLab/PATOWAS. We are committed to maintaining and improving the specific function modules per user comments and suggestions.

## Electronic supplementary material

Supplementary Information

Description of additional Supplementary Data

Supplementary Data 1

Supplementary Data 2

Supplementary Data 3

Supplementary Data 4

Supplementary Data 5

Supplementary Data 6

Supplementary Data 7

Supplementary Data 8

Supplementary Data 9

Supplementary Data 10

## Data Availability

All datasets, including presented case analysis data and results, are freely available at http://bioinfo.noble.org/PATOWAS/Download.gy.

## References

[1] Yang J, Lee SH, Goddard ME, Visscher PM (2011). GCTA: a tool for genome-wide complex trait analysis. Am. J. Human. Genet..

[2] Harper AL (2012). Associative transcriptomics of traits in the polyploid crop species *Brassica napus*. Nat. Biotech..

[3] Hindorff LA (2009). Potential etiologic and functional implications of genome-wide association loci for human diseases and traits. Proc. Natl Acad. Sci. USA.

[4] Pandey A (2012). Epistasis network centrality analysis yields pathway replication across two GWAS cohorts for bipolar disorder. Transl. Psychiatry.

[5] Carlborg O, Haley CS (2004). Epistasis: too often neglected in complex trait studies?. Nat. Rev. Genet..

[6] Eichler EE (2010). Missing heritability and strategies for finding the underlying causes of complex disease. Nat. Rev. Genet..

[7] Xu, S. Mapping quantitative trait loci by controlling polygenic background effects. *Genetics*, 10.1534/genetics.113.157032 (2013). 10.1534/genetics.113.157032 PMC3832267 24077303

[8] Zhang W, Dai X, Wang Q, Xu S, Zhao PX (2016). PEPIS: a pipeline for estimating epistatic effects in quantitative trait locus mapping and genome-wide association studies. PLoS Comput. Biol..

[9] Orgogozo, V., Morizot, B. & Martin, A. The differential view of genotype–phenotype relationships. *Front. Genet.* **6**, 10.3389/fgene.2015.00179 (2015). 10.3389/fgene.2015.00179 PMC4437230 26042146

[10] Bhatia A (2014). Yeast growth plasticity is regulated by environment-specific multi-QTL interactions. G3: Genes| Genomes| Genet..

[11] Gerke J, Lorenz K, Ramnarine S, Cohen B (2010). Gene–environment interactions at nucleotide resolution. PLoS Genet..

[12] Lee JT, Taylor MB, Shen A, Ehrenreich IM (2016). Multi-locus genotypes underlying temperature sensitivity in a mutationally induced trait. PLoS Genet..

[13] Muir W, Nyquist W, Xu S (1992). Alternative partitioning of the genotype-by-environment interaction. TAG Theor. Appl. Genet..

[14] Matsui T, Ehrenreich IM (2016). Gene-environment interactions in stress response contribute additively to a genotype-environment interaction. PLoS Genet..

[15] Patti GJ, Yanes O, Siuzdak G (2012). Innovation: Metabolomics: the apogee of the omics trilogy. Nat. Rev. Mol. Cell Biol..

[16] Bylesjö M, Eriksson D, Kusano M, Moritz T, Trygg J (2007). Data integration in plant biology: the O2PLS method for combined modeling of transcript and metabolite data. Plant J..

[17] Baye TM, Abebe T, Wilke RA (2010). Genotype–environment interactions and their translational implications. Pers. Med..

[18] Lu, G. et al. Associative transcriptomics study dissects the genetic architecture of seed glucosinolate content in *Brassica napus*. *DNA Res.*, 10.1093/dnares/dsu024 (2014). 10.1093/dnares/dsu024 PMC4263295 25030463

[19] Lin W, Feng R, Li H (2015). Regularization methods for high-dimensional instrumental variables regression with an application to genetical genomics. J. Am. Stat. Assoc..

[20] Chakraborty S, Ghosh M, Mallick BK (2012). Bayesian nonlinear regression for large p small n problems. J. Multivar. Anal..

[21] Diao G, Vidyashankar AN (2013). Assessing genome-wide statistical significance for large p small n problems. Genetics.

[22] Xu S, Zhu D, Zhang Q (2014). Predicting hybrid performance in rice using genomic best linear unbiased prediction. Proc. Natl. Acad. Sci. USA.

[23] Xu S (2017). Predicted residual error sum of squares of mixed models: an application for genomic prediction. G3: Genes|Genomes|Genetics.

[24] Bradbury PJ (2007). TASSEL: software for association mapping of complex traits in diverse samples. Bioinformatics.

[25] Chang, C. C., Chow, C. C., Tellier, L. C., Vattikuti, S., Purcell, S. M., & Lee, J. J. Second-generation PLINK: rising to the challenge of larger and richer datasets. *GigaScience* **4**, 10.1186/s13742-015-00 (2015). 10.1186/s13742-015-0047-8 PMC4342193 25722852

[26] Gibson G (2010). Hints of hidden heritability in GWAS. Nat. Genet..

[27] Cheng, J. et al. Identification and characterization of quantitative trait loci for shattering in Japonica Rice Landrace Jiucaiqing from Taihu Lake Valley, China. *Plant Genome* **9**, 10.3835/plantgenome2016.03.0034 (2016). 10.3835/plantgenome2016.03.0034 27902802

[28] Zhang K (2016). Down-regulation of OsSPX1 caused semi-male sterility, resulting in reduction of grain yield in rice. Plant Biotechnol. J..

[29] Hori K, Matsubara K, Yano M (2016). Genetic control of flowering time in rice: integration of Mendelian genetics and genomics. Theor. Appl. Genet..

[30] Harrop TWR (2016). Gene expression profiling of reproductive meristem types in early rice inflorescences by laser microdissection. Plant J..

[31] Ashikari M (2005). Cytokinin oxidase regulates rice grain production. Science.

[32] Yeh SY (2015). Down-regulation of cytokinin oxidase 2 expression increases tiller number and improves rice yield. Rice.

[33] Fadista J, Manning AK, Florez JC, Groop L (2016). The (in)famous GWAS P-value threshold revisited and updated for low-frequency variants. Eur. J. Hum. Genet..

[34] Xu S, Xu Y, Gong L, Zhang Q (2016). Metabolomic prediction of yield in hybrid rice. Plant J..

[35] Mitchell P, Sheehy JE (2006). Supercharging rice photosynthesis to increase yield. New Phytol..

[36] Chandra S (2014). Assessment of total phenolic and flavonoid content, antioxidant properties, and yield of aeroponically and conventionally grown leafy vegetables and fruit crops: a comparative study. Evid.-Based Complement. Altern. Med..

[37] Slatkin M (2008). Linkage disequilibrium—understanding the evolutionary past and mapping the medical future. Nat. Rev. Genet..

[38] Li J, Wei H, Liu T, Zhao PX (2014). GPLEXUS: enabling genome-scale gene association network reconstruction and analysis for very large-scale expression data. Nucleic Acids Res..

[39] Bino RJ (2004). Potential of metabolomics as a functional genomics tool. Trends Plant Sci..

[40] Lynn KS (2015). Metabolite identification for mass spectrometry-based metabolomics using multiple types of correlated ion information. Anal. Chem..

[41] Xie W (2010). Parent-independent genotyping for constructing an ultrahigh-density linkage map based on population sequencing. Proc. Natl. Acad. Sci. USA.

[42] Zhang W (2014). MET-COFEA: a liquid chromatography/mass spectrometry data processing platform for metabolite compound feature extraction and annotation. Anal. Chem..

[43] Zhang W, Lei Z, Huhman D, Sumner LW, Zhao PX (2015). MET-XAlign: a metabolite cross-alignment tool for LC/MS-based comparative metabolomics. Anal. Chem..

[44] Zhang, W. & Zhao, P. X. Quality evaluation of extracted ion chromatograms and chromatographic peaks in liquid chromatography/mass spectrometry-based metabolomics data. *BMC Bioinform.* **15**, 1471-2105-S11-S5 (2014). 10.1186/1471-2105-15-S11-S5 PMC4251050 25350128

[45] Hua JP (2002). Genetic dissection of an elite rice hybrid revealed that heterozygotes are not always advantageous for performance. Genetics.

[46] Hua J (2003). Single-locus heterotic effects and dominance by dominance interactions can adequately explain the genetic basis of heterosis in an elite rice hybrid. Proc. Natl. Acad. Sci. USA.

[47] Wang, J. et al. An expression quantitative trait loci-guided co-expression analysis for constructing regulatory network using a rice recombinant inbred line population. *J. Exp. Bot.* **65**, 1069–1079 (2014). 10.1093/jxb/ert464 PMC3935569 24420573

[48] Gong L (2013). Genetic analysis of the metabolome exemplified using a rice population. Proc. Natl. Acad. Sci. USA.

