# Supplementary material for: 2D association and integrative omics analysis in rice provides systems biology view in trait analysis
Source: Commun Biol. 2018 Sep 27;1:153. doi: 10.1038/s42003-018-0159-7 (PMC6160469; doi:10.1038/s42003-018-0159-7)
Supplement: Supplementary file 1 — Supplementary Information [file 42003_2018_159_MOESM1_ESM.pdf]

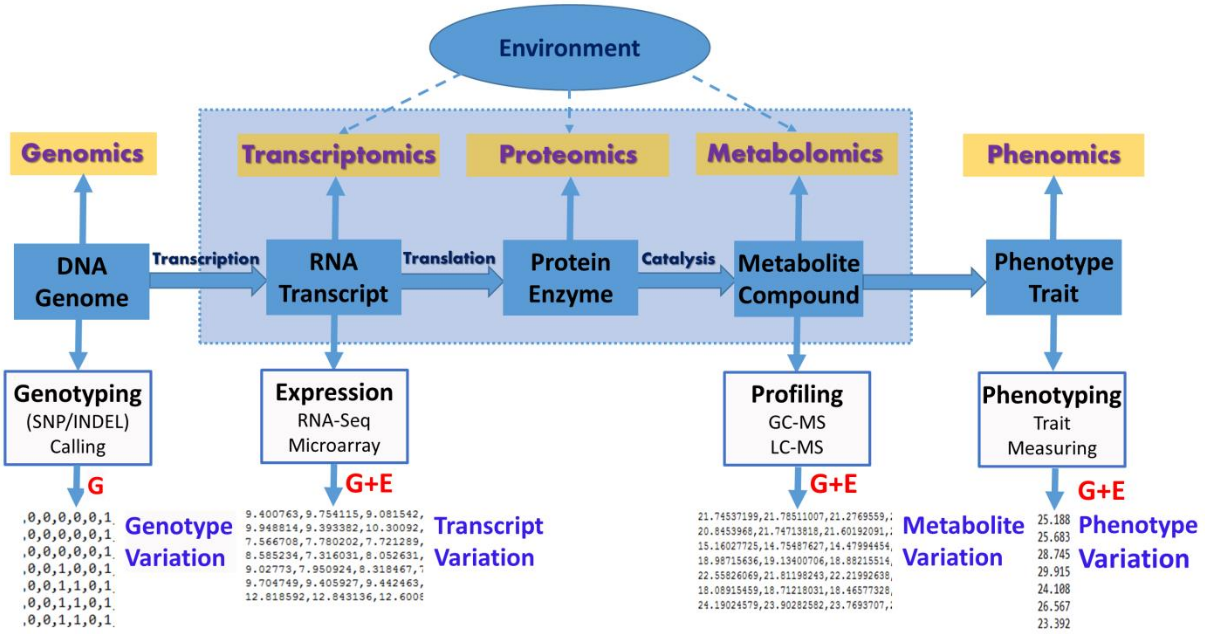

Supplementary Figure 1. System biological concept of the cascaded information from genotype and environment to phenotype, and the measurement at different omics levels.

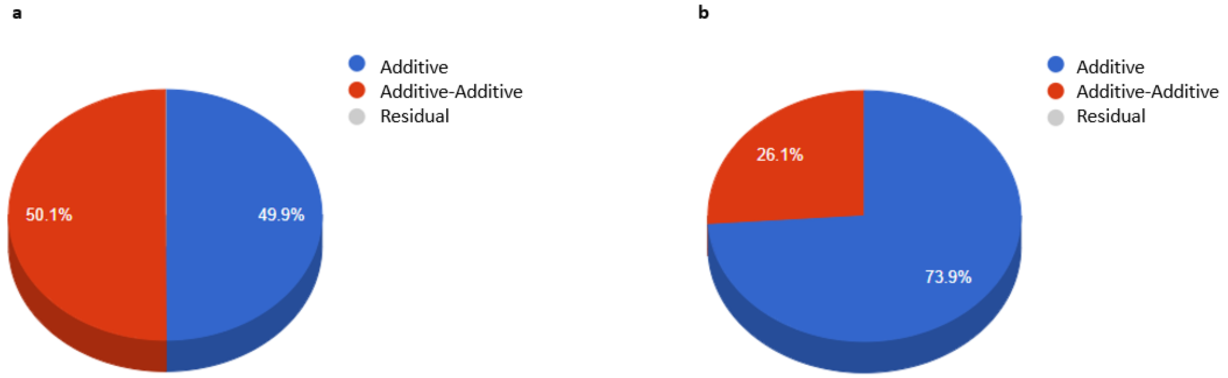

Supplementary Figure 2. Pie chart of variance component analysis result for trait YIELD (**a**) and KGW (**b**). The two analyses are based on the reduced 1,453 expressed transcripts.

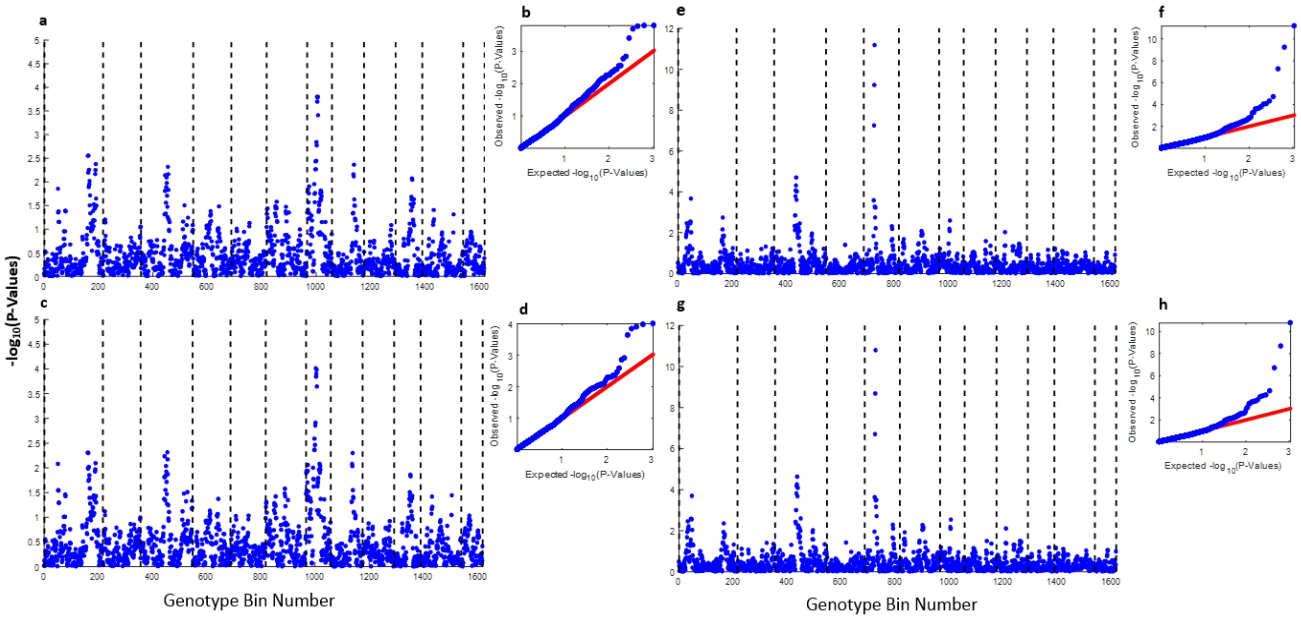

Supplementary Figure 3. Comparisons of 1D association mapping results, illustrated as the Manhattan plots (**a, c, e, g**) and Q-Q plots (**b, d, f, h**) for trait YIELD (**a-d**) and KGW (**e-h**), by PATOWAS (**a, b, e, f**) and TASSEL (**c, d, g, h**) respectively.

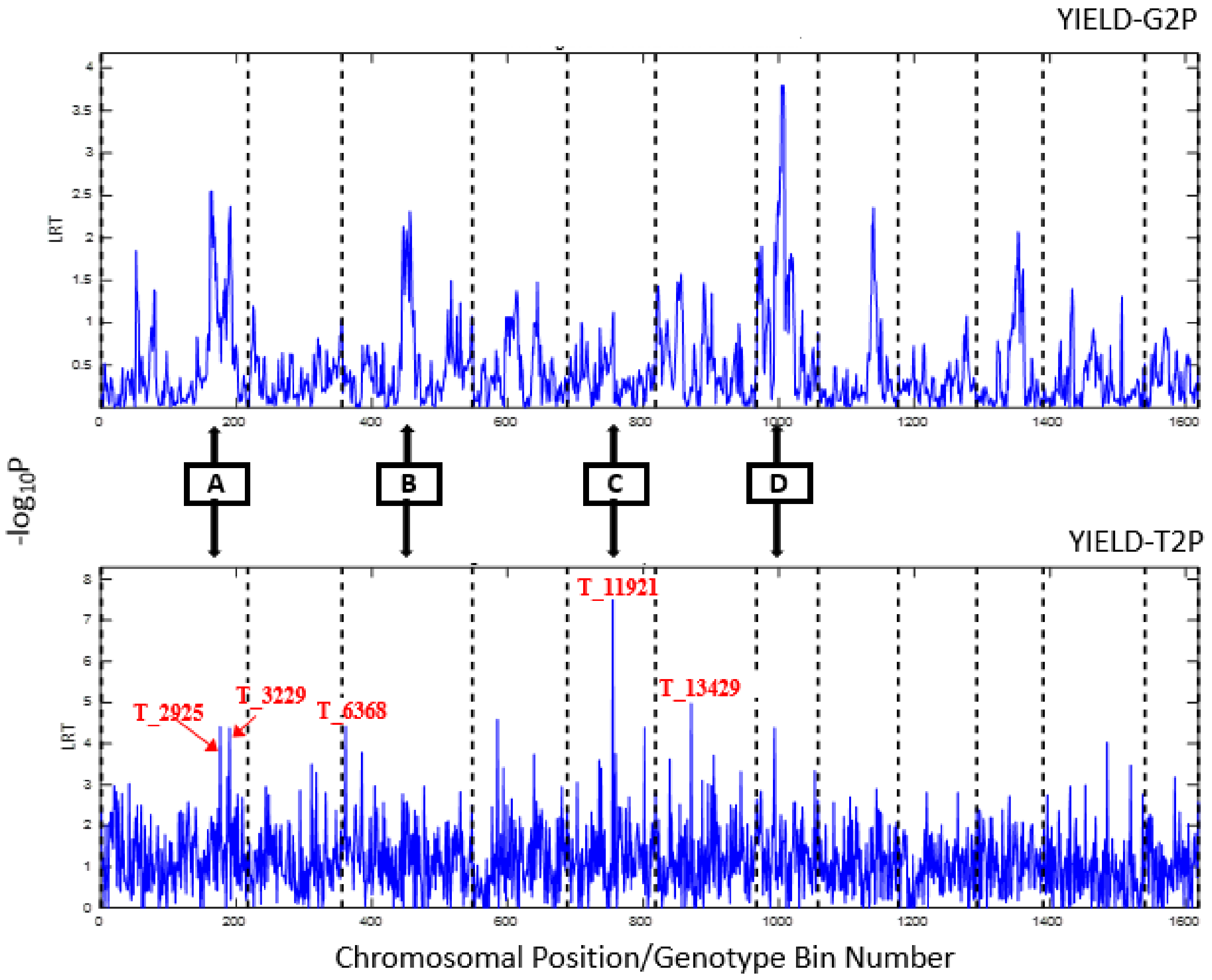

Supplementary Figure 4. Plots of 1D G2P and the aligned T2P association mapping results. The positional labeled five expressed genes (in red text) are literature reported to affect the grain yield. The four regions, either as high genotype variation region or high gene expression variation region, are marked. **Region A**: the high genotype variation and high gene expression variation region; **Region B**: high genotype variation but low gene expression region; **Region C**: low genotype variation but high gene expression variation region; **Region D**: high genotype variation and moderate gene expression region.

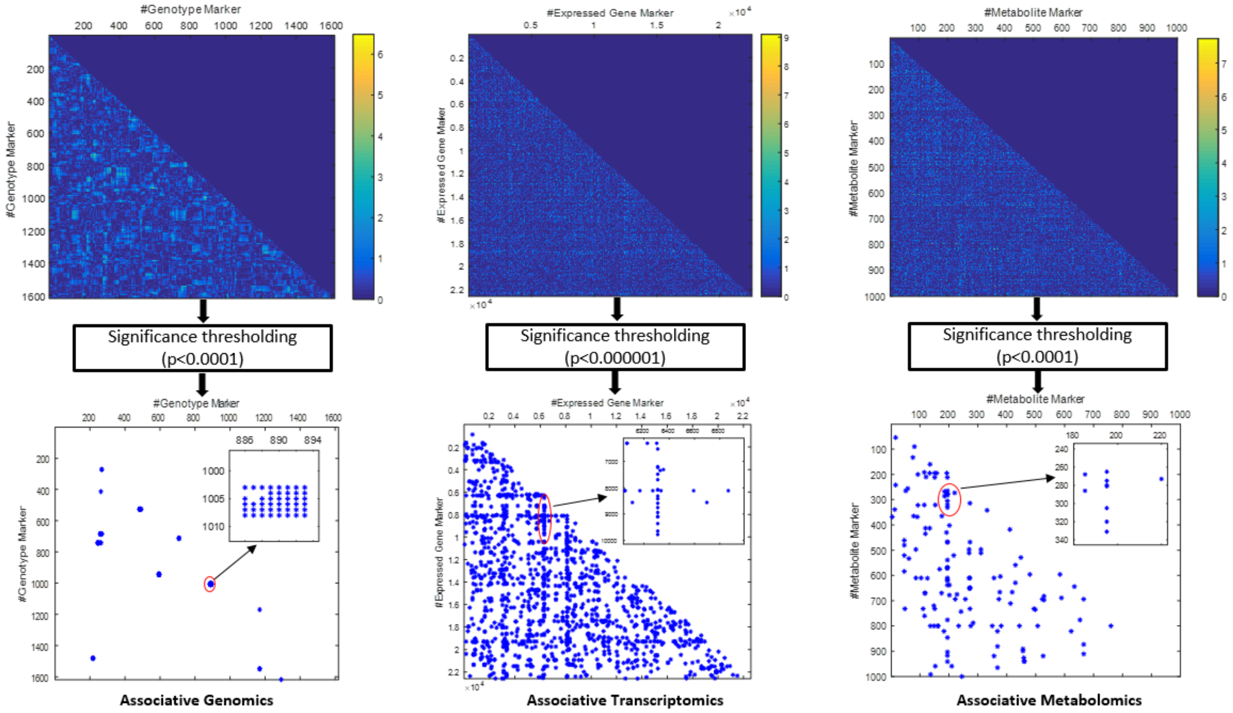

Supplementary Figure 5. The 2D association mapping and binary image illustration of the significant omics marker pair across three associative omics. Specified local regions in the binary image are zoomed in to illustrate the local structure pattern.

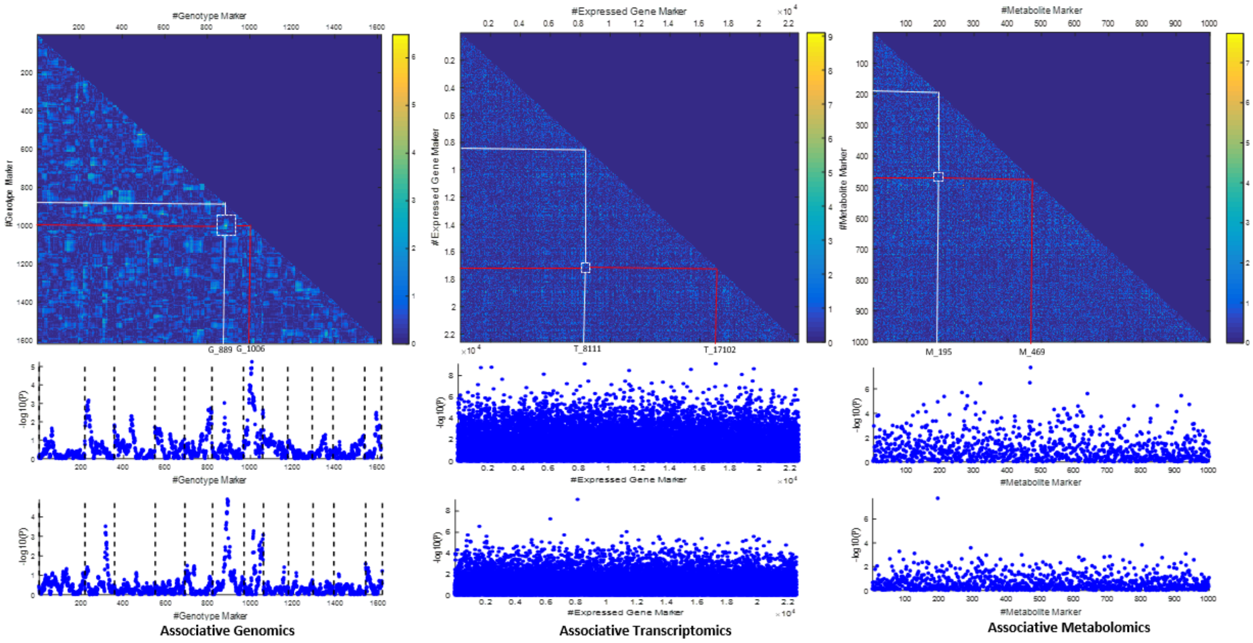

Supplementary Figure 6. 2D association mapping and the scattering plot of the conditional 1D association mappings for the representative marker pairs. The color scaling bar maps the $-log_{10}(P)$ value from low (deep blue) to high (yellow). Three representative omics marker pairs (*G_889, G_1006*), (*T_8111, T_17102*), *(M_195, M_469*) are pinpointed, and the two conditional 1D association line (white and red lines) for each marker pair are marked.

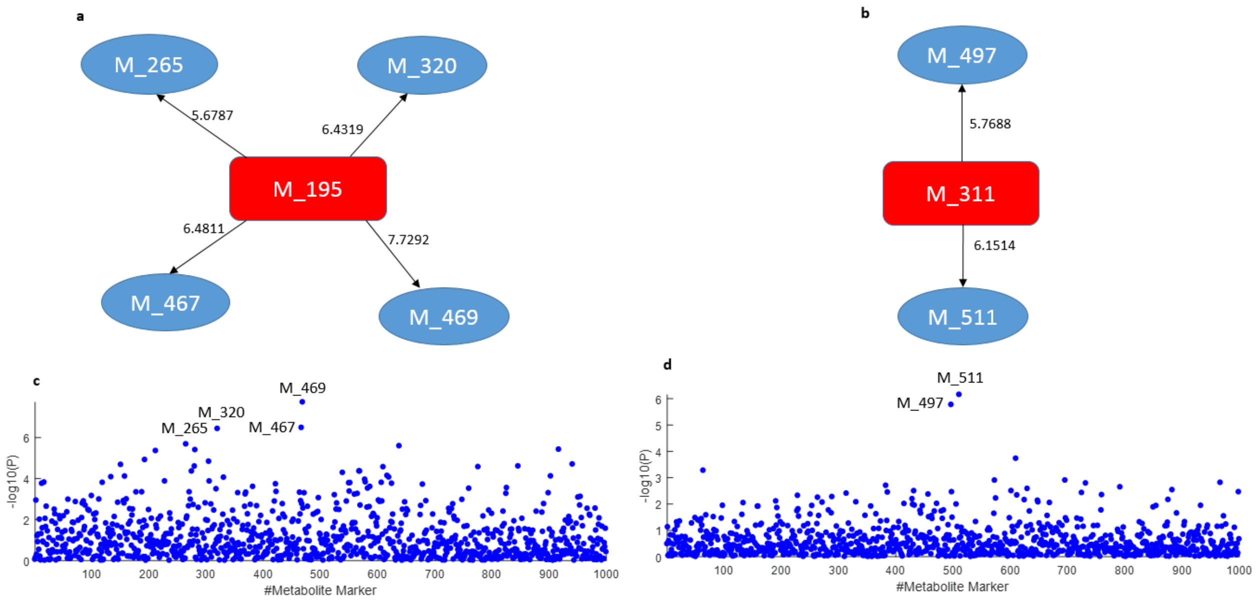

Supplementary Figure 7. Metabolite association network based on 6 of the top 10 significant metabolite marker pairs. The metabolite association networks consist of two subnetworks. **a** One subnetwork contains four pairs and is centered on hub marker M_195. **b** The other subnetwork contains two pairs and is centered on hub marker M_311. **c-d** Scatter plots of conditional 1D association mapping for the metabolite marker M_195 and M_311, respectively.

Supplementary Table 1. The literature retrieval and function annotation of top 10 expressed genes that show high associative significance with rice yield. The genes that have been found by authors to be reported in literature are highlighted.

Transcript Marker INDEX	Gene Locus ID	Genotype_Bin#	Significance	Annotated Gene Function to affect YIELD
T_2925	LOC_Os01g62860	176	4.4098	Seed shattering[1] (Cheng, He et al. 2016)
T_3229	LOC_Os01g67580	190	4.3748	Multidrug resistance-associated protein 9.[2]
T_6368	LOC_Os03g03070	361	4.4197	AGL20/SOC1, heading date, flowering time, transcription factor active in flowering time control[3].
T_9470	LOC_Os04g21130	585	4.5909	---
T_9471	LOC_Os04g21130	585	3.9589	---
T_11921	LOC_Os05g31040	755	7.5309	CKX9/Cytokinin dehydrogenase precursor; Catalyzes the oxidation of cytokinins, a family of N(6)-substituted adenine derivatives that are plant hormones[4]. CKX9 is expressed in rice shoot specifically, and down-regulation of CKX9 leads to accumulation of cytokinin, leading to increased tiller numer and rice yield[4,5].
T_12671	LOC_Os05g48390	802	4.3942	---
T_13429	LOC_Os06g11330	871	4.9773	MADS55 Interacting with FLC and repressing FT and SOC1, leading to delayed flowering[6]
T_15062	LOC_Os07g10130	994	4.3815	---
T_21146	LOC_Os11g35425	1484	4.029	---

Supplementary Table 2. The identification and classification of top 10 metabolites that show high associative significance with rice yield.

Metabolite Marker INDEX	Significance	Tissue	Metabolite name	Classification
M_15	2.8541	Leaf	Trigonelline	Others
M_23	2.8044	Leaf	L-Methionine	Amino acid
M_94	3.4404	Leaf	sn-Glycero-3-phosphocholine	Phosphatidylcholine
M_383	2.8505	Leaf	2'', 6''-O-Diacetyloninin	Flavonoid
M_386	3.6683	Leaf	Unknown	---
M_401	3.0565	Leaf	Unknown	---
M_453	2.9774	Leaf	Unknown	---
M_564	3.7358	Leaf	Unknown	---
M_643	3.5115	Leaf	Chrysoeriol	Flavonoid
M_691	3.0904	Seed	Unknown	---

Supplementary Table 3. Annotation of top 10 expressed gene transcript pairs that show high associative significance with rice yield. The annotation results are based on (https://string-db.org/overview.39947.html)

Expressed Transcript Marker Pair		Transcript Marker X				Transcript Marker Y			
(T_X,T_Y)	Significance	T_X	Gene Locus ID	Genotype Bin#	Annotated Gene Function	T_Y	Gene Locus ID	Genotype Bin#	Annotated Gene Function
(750 20421)	8.624	T_750	LOC_Os01g13470	51	KH domain containing protein, putative, expressed	T_20421	LOC_Os11g03230	1398	Nucleoside-triphosphatase, putative, expressed
(1524 8111)	8.725	T_1524	LOC_Os01g38610	103		T_8111	LOC_Os03g45280	466	Dehydrin, putative, expressed
(2228 8111)	8.7621	T_2228	LOC_Os01g52050	140	Growth, Systemin receptor SR160 precursor, putative, expressed	T_8111	LOC_Os03g45280	466	Dehydrin, putative, expressed
(5078 16273)	8.9161	T_5078	LOC_Os02g38480	311	Expressed protein	T_16273	LOC_Os07g48100	1058	CAMK includes calcium/calmodulin depedent protein kinases, expressed; CIPK serine-threonine protein kinases interact with CBL proteins
(5748 14716)	8.6253	T_5748	LOC_Os02g51100	324	Arsenical pump-driving ATPase, putative, expressed	T_14716	LOC_Os07g02920	979	DUF538 domain containing protein, putative, expressed
(8111 8406)	9.0821	T_8111	LOC_Os03g45280	466	Dehydrin, putative, expressed	T_8046	LOC_Os03g51330	503	Brassinosteroid, plant hormone
(8111 17102)	9.0937	T_8111	LOC_Os03g45280	466	Dehydrin, putative, expressed	T_17102	LOC_Os08g26220	1119	---
(8111 19626)	8.6186	T_8111	LOC_Os03g45280	466	Dehydrin, putative, expressed	T_19626	LOC_Os10g29620	1348	Tyrosine protein kinase domain containing protein, putative, expressed
(8555 16807)	8.7353	T_8555	LOC_Os03g53770	520	---	T_16807	LOC_Os08g10550	1102	Potassium transporter, putative, expressed; High-affinity potassium transporter
(8555 16808)	8.7353	T_8555	LOC_Os03g53770	520	---	T_16808	LOC_Os08g10550	1103	Potassium transporter, putative, expressed; High-affinity potassium transporter

Supplementary Table 4. The identification and classification of top 10 metabolite pairs that show high associative significance with rice YIELD.

Metabolite Marker Pair		Metabolite Marker X				Metabolite Marker Y			
**(M_X, M_Y)**	**Significance**	**M_X**	**Tissue**	**Metabolite name**	**Classification**	**M_Y**	**Tissue**	**Metabolite name**	**Classification**
(413 134)	5.8548	M_413	Leaf	Unknown	---	M_134	Leaf	Unknown	---
(800 163)	5.8317	M_800	Seed	Unknown	---	M_163	Leaf	Buergeriside C1	Polyphenol
(265 195)	5.6787	M_265	Leaf	Unknown	---	M_195	Leaf	Unknown	---
(320 195)	6.4319	M_320	Leaf	Unknown	---	M_195	Leaf	Unknown	---
(467 195)	6.4811	M_467	Leaf	Unknown	---	M_195	Leaf	Unknown	---
(469 195)	7.7292	M_469	Leaf	Unknown	---	M_195	Leaf	Unknown	---
(610 270)	5.9456	M_610	Leaf	Unknown	---	M_270	Leaf	Apigenin 8-C-glucoside	Flavonoid
(497 311)	5.7688	M_497	Leaf	Tricin O-rutinoside	Flavonoid	M_311	Leaf	Chrysoeriol 5-O-hexoside	Flavonoid
(511 311)	6.1514	M_511	Leaf	Unknown	---	M_311	Leaf	Chrysoeriol 5-O-hexoside	Flavonoid
(881 368)	5.6891	M_881	Seed	Tricin derivative	Flavonoid	M_368	Leaf	Unknown	---

Supplementary References

1 Cheng, J. *et al.* Identification and Characterization of Quantitative Trait Loci for Shattering in Japonica Rice Landrace Jiucaiqing from Taihu Lake Valley, China. *The Plant Genome* **9**, (2016).

2 Zhang, K. *et al.* Down-regulation of OsSPX1 caused semi-male sterility, resulting in reduction of grain yield in rice. *Plant Biotechnology Journal* **14**, 1661-1672, (2016).

3 Hori, K., Matsubara, K. & Yano, M. Genetic control of flowering time in rice: integration of Mendelian genetics and genomics. *Theoretical and Applied Genetics* **129**, 2241-2252, (2016).

4 Ashikari, M. *et al.* Cytokinin Oxidase Regulates Rice Grain Production. *Science* **309**, 741 (2005).

5 Yeh, S.-Y. *et al.* Down-Regulation of Cytokinin Oxidase 2 Expression Increases Tiller Number and Improves Rice Yield. *Rice* **8**, 36, (2015).

6 Harrop, T. W. R. *et al.* Gene expression profiling of reproductive meristem types in early rice inflorescences by laser microdissection. *The Plant Journal* **86**, 75-88, (2016).
