# Supplementary material for: 2D association and integrative omics analysis in rice provides systems biology view in trait analysis
Source: Commun Biol. 2018 Sep 27;1:153. doi: 10.1038/s42003-018-0159-7 (PMC6160469; doi:10.1038/s42003-018-0159-7)
Supplement: Supplementary file 2 — Description of additional Supplementary Data [file 42003_2018_159_MOESM2_ESM.docx]

**Description of Additional Supplementary Items:**

**Supplementary Data 1** - Field phenotypic trait data for yield (YIELD) as a vector of 210$\times$1.

(.csv)

**Supplementary Data 2** - Field phenotypic trait data for (kilo-)thousand grain weight (KGW) as a vector of 210$\times$1.

(.csv)

**Supplementary Data 3** - Genotype data as a matrix of 1,619$\times$210.

(.csv)

**Supplementary Data 4** - Gene expression transcript data as a matrix of 22,584$\times$210.

(.csv)

**Supplementary Data 5** - Metabolite data as a matrix of 1,000$\times$210.

(.csv)

**Supplementary Data 6** - Mapping of expressed genes to genotype bins.

Of the original 24,994 expressed genes, 22,584 genes were mapped to 1,619 genotype bins.

(.xlsx)

**Supplementary Data 7** – reduced gene expression data to trait YIELD as a matrix of 1,543$\times$ 210.

(.csv)

**Supplementary Data 8** – reduced gene expression data to trait KGW as a matrix of 1,543$\times$ 210.

(.csv)

**Supplementary Data 9** – $R^{2}$ values of the expressed genes/transcripts. The predictabilities (PREDs) are predicted from the genotype data by the HAT method.

(.xlsx)

**Supplementary Data 10** – $R^{2}$ values of the metabolites. The predictabilities (PREDs) are predicted from the genotype data by the HAT method.

(.xlsx)
